# Treating without guidelines: management and outcomes of lung cancer diagnosed during pregnancy — a systematic review

**DOI:** 10.3389/fonc.2026.1780320

**Published:** 2026-05-13

**Authors:** Nehemias Guevara Rodriguez, Noemy Coreas, Coral Olazagasti, Martina Imbimbo, Narjust Florez

**Affiliations:** 1 Department of Internal Medicine, Division of Hematology, Oncology, and Bone Marrow Transplantation, SSM Health–Saint Louis University School of Medicine, St. Louis, MO, United States; 2 Department of Gynecology and Obstetrics, Gynecologic Oncology, Universidad de El Salvador (UES); Instituto Salvadoreño del Seguro Social (ISSS), San Salvador, El Salvador; 3 Department of Medicine, Division of Hematology and Oncology, Sylvester Comprehensive Cancer Center, University of Miami Miller School of Medicine, Miami, FL, United States; 4 Oncology Institute of Southern Switzerland, Ente Ospedaliero Cantonale, Ospedale Regionale Bellinzona e Valli, Bellinzona, Switzerland; 5 Department of Medical Oncology, Dana-Farber Cancer Institute, Harvard Medical School, Boston, MA, United States

**Keywords:** chemotherapy, fetal outcomes, lung cancer, non–small cell lung cancer, pregnancy, systematic review, targeted therapy

## Abstract

**Background:**

Lung cancer during pregnancy is exceedingly rare and poses unique diagnostic and therapeutic challenges. Clinical decisions must balance urgent, potentially lifesaving maternal treatment with fetal safety, often in the absence of evidence-based guidelines. This systematic review aimed to characterize clinical features, treatment patterns, and maternal–fetal outcomes among reported cases of primary lung cancer diagnosed during pregnancy, focusing on case reports and case series.

**Methods:**

We systematically searched PubMed, Embase, Scopus, and Google Scholar from 1948 through March 2024 for articles reporting primary lung cancer diagnosed during pregnancy. Eligible studies included case reports, case series, or observational reports providing individual-level data. We extracted demographics, tumor characteristics, staging, molecular profile, treatment type and timing (during pregnancy and postpartum), and maternal–fetal outcomes. Descriptive statistics were performed, and exploratory analyses were conducted to assess associations between selected variables and outcomes.

**Results:**

A total of 4,411 records were identified, and 88 publications contributing 96 unique cases met the inclusion criteria. The mean maternal age at diagnosis was 32.9 ± 5.0 years (range 18–42). Most patients (65%) presented with stage III–IV disease at diagnosis. Tobacco use was reported in 33% of cases and was significantly associated with advanced stage at presentation (p = 0.0006). Among tumors with reported molecular testing, 58.9% harbored actionable driver alterations, most commonly ALK rearrangements (32.9%) and EGFR mutations (21.4%). Chemotherapy during pregnancy was administered in 22.8% of cases, most commonly platinum-based doublets, with a mean gestational age at initiation of 21.8 weeks (SD ± 10.4). Targeted therapies, including EGFR and ALK tyrosine kinase inhibitors, were typically initiated in the postpartum period in 30 patients (31.6% of the overall cohort). Among reported pregnancies, 69 (71.9%) resulted in live births. Term delivery occurred in 46.9% of cases, while preterm delivery occurred in 25.0%. Pregnancy termination was reported in 17.7% of cases and fetal demise in 1.0%. Advanced maternal disease stage was significantly associated with adverse fetal outcomes, including preterm delivery, pregnancy termination, or fetal demise (p < 0.0001).

**Conclusion:**

Lung cancer during pregnancy most often presents at advanced stages, with a high prevalence of actionable molecular alterations. Multidisciplinary management and routine molecular profiling are essential. In the limited case-based literature, chemotherapy administered during the second and third trimesters has not demonstrated a clear signal of major congenital toxicity; however, these findings should be interpreted with caution, given the small sample size, heterogeneity of reporting, and lack of long-term follow-up. Targeted therapies are generally deferred until the postpartum period. Early diagnosis, optimized imaging protocols, and coordinated maternal–fetal care are critical. International registries and consensus guidelines are urgently needed to guide treatment decisions and improve maternal and fetal outcomes in this rare clinical scenario.

## Introduction

Cancer during pregnancy is rare, affecting approximately 1 in 1,000 pregnancies, but its incidence is expected to rise as more individuals delay childbearing into their thirties and forties (1–3). Lung cancer is the leading cause of cancer-related death worldwide and increasingly affects younger adults, including women of reproductive age (4). When lung cancer is diagnosed during an ongoing pregnancy, clinicians must navigate complex trade-offs between maternal benefit and fetal risk in the context of limited prospective data.

Lung cancer during pregnancy remains poorly characterized. Most of the available evidence consists of single case reports and small series published over several decades (5–8). Older literature often lacks uniform staging, detailed treatment descriptions, or long-term follow-up (5–7). More recent pooled analyses and registry-based data have begun to describe patterns of presentation, molecular features, and outcomes in pregnancy-associated lung cancer, but these cohorts remain modest in size and heterogeneous in reporting (9, 10). Expert perspectives emphasize the need for structured, multidisciplinary care models that bridge thoracic oncology and obstetrics to address these unique clinical scenarios (11).

Diagnostic evaluation is frequently delayed or modified in pregnant patients with cancer because of concerns about fetal radiation exposure and procedural risks. Historically, these concerns have limited the use of chest CT and nuclear imaging in pregnant patients with suspected lung cancer. However, contemporary guidance from radiology and obstetric societies indicates that, with appropriate shielding and protocol optimization, many imaging modalities can be performed with fetal doses well below teratogenic thresholds (12–15). Despite this, pregnant patients may still experience delays in imaging and biopsy compared with nonpregnant peers, potentially contributing to later-stage diagnosis.

Systemic therapy decisions are further complicated by gestational age at diagnosis. First-trimester exposure to cytotoxic chemotherapy is associated with a higher risk of major congenital malformations and is generally avoided (2, 16, 17). In contrast, available cohort and registry data suggest that selected regimens, particularly platinum-based combinations, may be administered during the second and third trimesters with manageable obstetric and neonatal risks when carefully timed (2, 18–22). However, pharmacokinetic changes during pregnancy and long-term neurodevelopmental outcomes in exposed children remain incompletely understood (18, 19, 21, 23).

Over the past two decades, the treatment landscape for lung cancer has been transformed by molecular profiling and targeted therapies. Somatic alterations in EGFR, ALK, and other oncogenic drivers are key determinants of prognosis and treatment responsiveness (24–30). Younger, never-smoking patients, disproportionately women, are more likely to harbor actionable mutations and gene rearrangements (27, 30, 31). Modern first-line regimens increasingly incorporate tyrosine kinase inhibitors (TKIs) and immunotherapies, raising important questions regarding their role when lung cancer is diagnosed during pregnancy (24–30). Data on the safety of TKIs and immune checkpoint inhibitors in pregnancy remain limited to small series and pharmacovigilance reports, and most guidelines recommend deferring these agents until after delivery when feasible (13, 15, 21, 31, 32).

These clinical uncertainties intersect with broader disparities in lung cancer care. Prior population-based studies have demonstrated that Black, Hispanic, and socioeconomically marginalized populations are less likely to receive timely imaging, molecular testing, and guideline-concordant therapy and are more likely to present with advanced disease (33–36). While these disparities have not been directly evaluated within pregnancy-specific cohorts, they may further complicate care delivery in this setting. Emerging work in thoracic oncology highlights the importance of patient-centered care, including attention to motherhood, survivorship, and structural factors that influence access to care and clinical trial participation (37, 38).

Against this backdrop, we conducted a systematic review to synthesize the existing literature on lung cancer diagnosed during an ongoing pregnancy. Our objectives were to characterize patient demographics, tumor features (including molecular profiles), diagnostic pathways, treatment approaches (with specific attention to chemotherapy and targeted therapies), and maternal and fetal outcomes. By aggregating individual-level data across multiple decades and therapeutic eras, we aim to inform multidisciplinary decision-making, highlight gaps in evidence, and identify priorities for future research and guideline development.

## Methods

### Study design

We conducted a systematic review of the literature on primary lung cancer diagnosed during pregnancy in accordance with the Preferred Reporting Items for Systematic Reviews and Meta-Analyses (PRISMA 2020) guidelines. The review protocol prespecified the research question, eligibility criteria, data elements, and analytic approach.

The objective of this review was to evaluate clinical characteristics, diagnostic approaches, treatment patterns, and maternal–fetal outcomes among patients diagnosed with primary lung cancer during an ongoing pregnancy. The research question was structured according to a PICOS framework: population (pregnant patients with primary lung cancer), interventions (systemic therapy, surgery, radiotherapy, and supportive care), comparator (not applicable given the case-based design), outcomes (clinical characteristics, maternal outcomes, and fetal/neonatal outcomes), and study designs (case reports, case series, and observational studies with extractable individual-level data).

**Figure 1:**
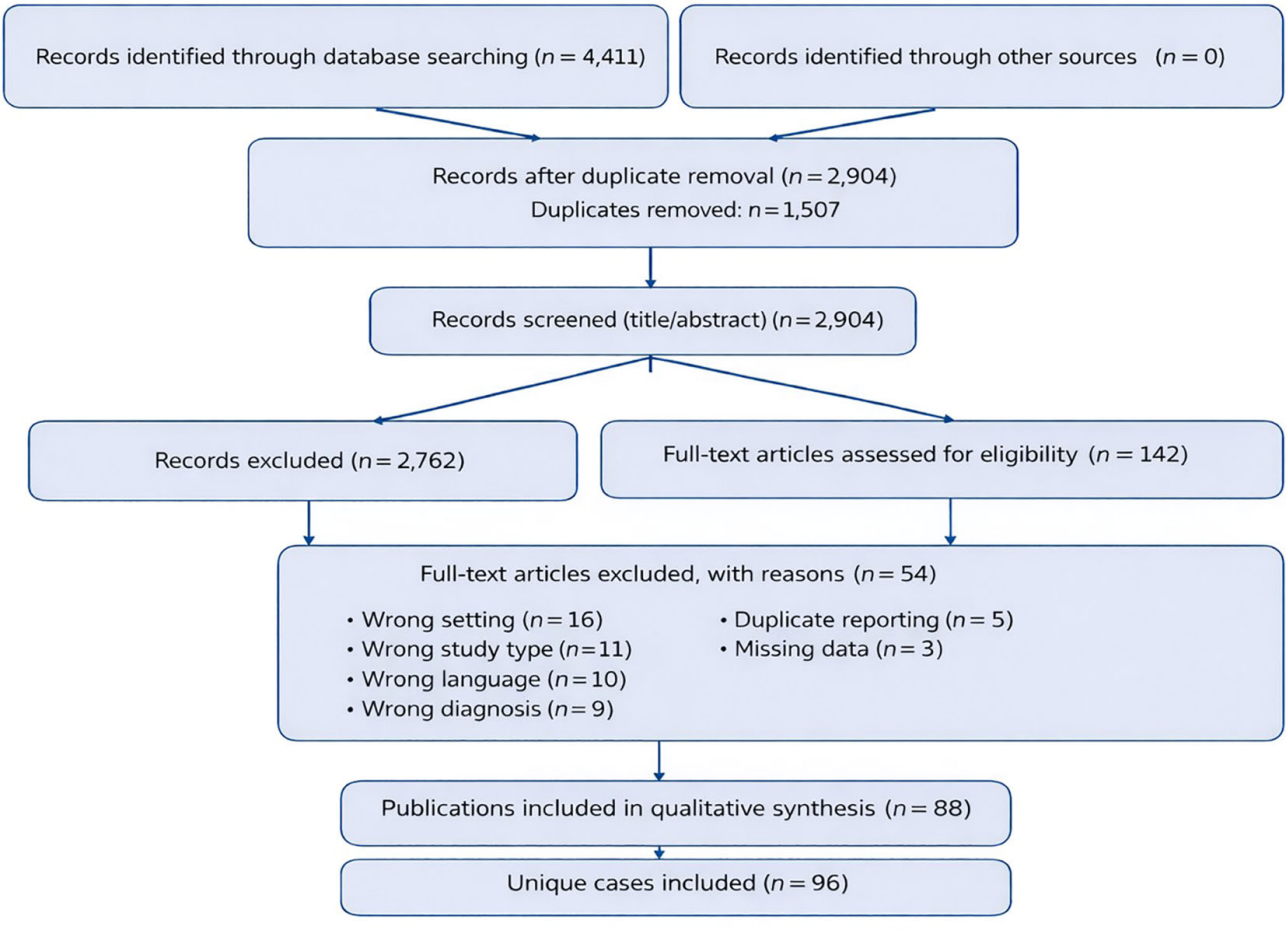
PRISMA 2020 flow diagram of study selection. Numbers represent records identified, screened, assessed for eligibility, and included in the qualitative synthesis.

Given the rarity of this condition and the absence of randomized or comparative studies, the review focused on case reports, case series, and observational studies reporting individual-level data. The study selection process is summarized in the PRISMA flow diagram (**Figure 1**).

### Data sources and search strategy

We performed a comprehensive literature search of PubMed, Embase, and Scopus from database inception (1948) through March 31, 2024. To capture additional case reports and small case series that may not be indexed in major databases, we also searched Google Scholar and manually reviewed the reference lists of all eligible studies and relevant review articles.

Search strategies were developed using combinations of controlled vocabulary (e.g., MeSH in PubMed and Emtree in Embase) and free-text keywords related to lung cancer and pregnancy. Core concepts included lung malignancy (e.g., “lung neoplasms,” “non–small cell lung cancer,” and “small cell lung cancer”) and pregnancy-related terms (e.g., “pregnancy,” “pregnant,” “gestation,” and “maternal”). Boolean operators and truncation were adapted for each database.

Database-specific filters and limits were applied as appropriate to optimize sensitivity for case reports and small case series. PubMed searches were limited to human studies and the English language. Embase searches were restricted to human studies, including articles, case reports, and case series. Scopus was searched without language restrictions at the search stage. Google Scholar results were screened manually, with the first 300 results reviewed to identify additional relevant cases. Language restrictions were applied at the eligibility stage, with inclusion limited to studies available in English or with sufficient English-language information to allow extraction of key clinical variables. Two reviewers independently screened titles, abstracts, and full-text articles for eligibility. Given the rarity of lung cancer during pregnancy and the case-based nature of the literature, the search strategy was designed to maximize sensitivity and capture all available reports across multiple decades. An updated literature check was performed through March 2026 to identify any newly published studies meeting the inclusion criteria and to assess whether they would materially alter the findings. Complete electronic search strategies for each database are provided in **Supplementary Table 1**.

### Eligibility criteria

Studies were eligible for inclusion if they reported cases of primary lung cancer of any histologic subtype diagnosed during an ongoing pregnancy. The population of interest consisted of pregnant patients diagnosed with primary lung cancer, and the outcomes of interest included clinical characteristics, treatment approaches, and maternal and/or fetal outcomes.

Eligible studies were required to provide individual-level clinical information, including at least one of the following variables: maternal age, gestational age at diagnosis, tumor histology, disease stage, treatment characteristics, or maternal and/or fetal outcomes. We included case reports, case series, and observational studies that allowed the extraction of patient-level data.

Studies were included if the full text was available in English or if sufficient data were available in the English abstract to allow extraction of key clinical variables. No restrictions were applied based on publication date.

We excluded publications describing pulmonary metastases from extrapulmonary primary malignancies without evidence of a primary lung cancer. Studies reporting lung cancer diagnosed only after delivery, without clear documentation that the diagnosis occurred during pregnancy, were also excluded. Narrative reviews, editorials, clinical guidelines, conference abstracts without extractable individual-level data, and animal or preclinical studies were excluded.

When multiple publications reported overlapping patient populations, study characteristics were carefully reviewed to identify potential duplication. In such cases, data were consolidated, and the report providing the most complete clinical information and the longest available follow-up was prioritized for inclusion.

### Study selection

The literature search identified 4,411 records. After removal of 1,507 duplicate records, 2,904 unique articles were screened by title and abstract. Two reviewers independently selected studies, and discrepancies were resolved through discussion.

Of the screened records, 2,762 were excluded for failing to meet the predefined inclusion criteria. A total of 142 full-text articles were assessed for eligibility, of which 54 were excluded for predefined reasons, including wrong setting, inappropriate study design, non-primary lung cancer diagnosis, duplicate reporting, or insufficient data.

Ultimately, 88 publications contributing 96 unique cases of primary lung cancer diagnosed during an ongoing pregnancy were included in the qualitative synthesis (**Figure 1**).

### Data extraction

A standardized data extraction form was developed and pilot tested on a subset of included studies to ensure consistency. Two reviewers independently extracted data from each eligible article, and discrepancies were resolved through discussion, with involvement of a third reviewer when necessary. Extracted data included maternal characteristics (age at diagnosis, gravidity and parity, and relevant comorbidities); pregnancy-related variables (gestational age and trimester at diagnosis, and singleton versus multiple gestation); and tumor features (histologic subtype, stage at diagnosis, presence and sites of metastases, and available molecular or biomarker data). We also collected information on diagnostic evaluation, including imaging modalities used, use of fetal shielding, and tissue diagnostic methods. Treatment details during pregnancy were recorded, including surgery, systemic therapy, and radiotherapy, along with gestational age at treatment initiation when specified. Postpartum management was documented, including systemic therapy, targeted therapy, immunotherapy, surgery, and radiotherapy. Maternal outcomes of interest included treatment response, disease progression, survival status at last follow-up, and time from diagnosis to last follow-up or death. Fetal and neonatal outcomes included gestational age at delivery, mode of delivery, birth weight, Apgar scores, congenital anomalies, fetal demise, neonatal complications, and follow-up status. Data on placental and fetal pathology were also extracted, including whether placental examination was performed and whether metastases were identified in the placenta or fetus. When reports provided incomplete or aggregated data, individual-level information was extracted when possible. Missing variables were recorded as not reported and were excluded from analyses requiring those specific data.

### Quality assessment

**Figure 2:**
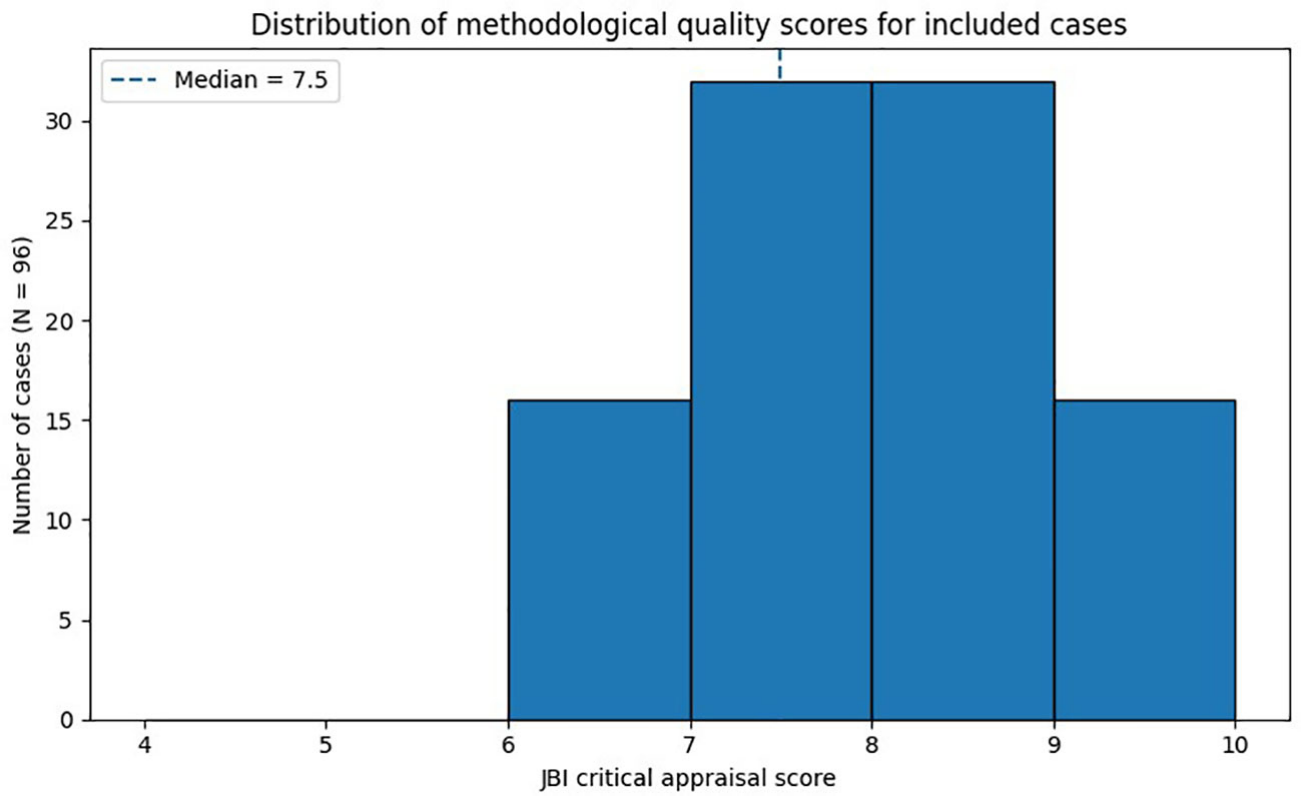
Distribution of methodological quality scores for included cases assessed using the Joanna Briggs Institute (JBI) critical appraisal checklist for case reports and case series. the median score was 7.5 (interquartile range 7–9), reflecting overall moderate to good reporting quality. Scores represent completeness of reporting and do not imply risk-of-bias weighting.

Given that the included studies consisted predominantly of case reports and case series, methodological quality was assessed using the Joanna Briggs Institute (JBI) Critical Appraisal Checklist for Case Reports and Case Series. This tool evaluates key domains, including clarity of the case definition and diagnostic confirmation, completeness of clinical and obstetric reporting, adequacy of the diagnostic evaluation, level of detail in the treatment description, and reporting of maternal and fetal outcomes. Each case report, or individual case within a case series when data were reported separately, was evaluated at the case level across the applicable JBI domains. Each item was assessed as “yes,” “no,” or “unclear,” and a summary score was calculated for each case based on the number of domains adequately reported. No modifications were made to the JBI tool. Summary scores were used descriptively to characterize the completeness and transparency of reporting and do not represent a formal quantitative measure of risk of bias. Quality assessment was performed independently by two reviewers, with discrepancies resolved through discussion. Quality scores were summarized descriptively to provide context for the overall strength and completeness of the available evidence and are presented in **Figure 2**. No studies were excluded based on quality assessment.

### Data synthesis and statistical analysis

Given the rarity of lung cancer during pregnancy and the clinical heterogeneity of reported cases, we performed a primarily descriptive synthesis. Continuous variables were summarized using means with standard deviations or medians with interquartile ranges, as appropriate. Categorical variables were reported as counts and percentages. Denominators varied depending on data availability for each variable. Follow-up time for survival analyses was defined as the interval from diagnosis to the last reported follow-up or death, as described in each case report. Survival outcomes were analyzed descriptively, and only cases with available follow-up data were included in each survival summary (complete-case approach). Denominators, therefore, varied depending on data availability for each survival outcome. Exploratory analyses were performed to examine associations between selected clinical variables and outcomes. Specifically, tobacco exposure (yes vs. no) was compared with disease stage at diagnosis (early stage [I–II] vs. advanced stage [III–IV]). In addition, maternal disease stage (early vs. advanced) was compared with pregnancy outcomes. For exploratory analysis of fetal outcomes, an adverse fetal outcome was defined as a composite of preterm delivery, pregnancy termination, or fetal demise, while term delivery was considered separately. Analyses were restricted to cases with available data for the variables of interest (complete-case analysis). Cases with missing data were excluded from the corresponding comparisons. Categorical comparisons were performed using chi-square tests when expected cell counts were sufficient and Fisher’s exact test when expected cell counts were small. Given the limited sample size and heterogeneity of case-based reports, all statistical analyses were considered exploratory and hypothesis-generating and were interpreted with caution. A two-sided p-value of <0.05 was considered statistically significant.

## Results

### Study selection and study characteristics

The literature search identified 4,411 records. After removal of 1,507 duplicate records, 2,904 unique articles were screened by title and abstract. Of these, 2,762 were excluded because they did not involve primary lung cancer diagnosed during pregnancy or lacked relevant clinical detail. A total of 142 full-text articles were assessed for eligibility, of which 54 were excluded for predefined reasons. Ultimately, 88 publications contributing 96 unique cases of primary lung cancer diagnosed during an ongoing pregnancy were included in the final analysis. The study selection process is shown in the PRISMA flow diagram (**Figure 1**).

Most included publications consisted of single case reports or small case series published over several decades and across evolving therapeutic eras (**Table 1**), with variable reporting quality. Using the Joanna Briggs Institute (JBI) Critical Appraisal Checklist for Case Reports and Case Series, the median quality score was 7.5 (interquartile range 7–9), reflecting overall moderate to good completeness of clinical and obstetric reporting (**Figure 2**).

**Table 1:** Publication year and therapeutic era of reported cases.

Therapy era	Approx. years	Therapeutic context	Representative agents/milestones	Number of reported cases (n)	% of total
Pre-platinum era	1948–1979	Supportive care; diagnosis often delayed or post-mortem	—	5	5%
Platinum-based chemotherapy era	1980–2003	Introduction of platinum-based combination chemotherapy	Cisplatin, Carboplatin, Etoposide	12	13%
Early targeted-therapy era (EGFR)	2004–2011	Discovery of EGFR mutations; first-generation TKIs	Gefitinib, Erlotinib	10	10%
ALK-directed and second-generation TKI era	2012–2016	Identification of ALK rearrangements; expansion of targeted therapy	Crizotinib, Ceritinib	14	15%
Immunotherapy era	2017–2020	Introduction of immune checkpoint inhibitors in lung cancer	Pembrolizumab, Nivolumab	18	19%
Modern targeted + immunotherapy era	2021–2024	Next-generation TKIs and widespread molecular profiling	Alectinib, Osimertinib, other TKIs; combined immunotherapy + chemotherapy	37	38%
Total	1948–2024	—	—	96	100%

EGFR, epidermal growth factor receptor; ALK, anaplastic lymphoma kinase; TKI, tyrosine kinase inhibitor; IO, immunotherapy. Therapy eras are defined based on the predominant systemic treatment landscape at the time of case publication. In some cases, diagnosis and treatment spanned multiple eras; cases were classified according to the dominant therapeutic context. These categories reflect evolving treatment paradigms and do not imply uniform access to or use of specific therapies across all reported cases.

### Patient demographics and clinical features

Across the 96 cases, the mean maternal age at diagnosis was 32.9 years (standard deviation 5.0; range 18–42), with most patients diagnosed in their thirties. Gestational age at diagnosis was reported for 91 pregnancies. The mean gestational age was 24.1 weeks (standard deviation 8.9; range 5–39). Diagnoses most commonly occurred in the second trimester, followed by the third, while first-trimester diagnoses were less frequent. Smoking status was available for 76 patients (79.2% of the cohort). Among these, 25 (32.9%) were current or former smokers, and 51 (67.1%) were never smokers. In exploratory analyses restricted to cases with available data, tobacco exposure was significantly associated with advanced-stage disease at diagnosis (stage III–IV vs. I–II; p = 0.0006). Presenting symptoms were reported in 79 cases (82.3%), most commonly cough, dyspnea, chest pain, hemoptysis, and fatigue. In several reports, initial symptoms were attributed to pregnancy-related physiologic changes, contributing to delays in imaging and definitive diagnosis. Baseline demographic and clinical characteristics are summarized in **Table 2**.

**Table 2:** Demographic and clinical characteristics of included patients (N = 96).

Variable	Value
Mean maternal age (years)	32.9 ± 5.0
Age range (years)	18–42
Stage III or IV at diagnosis (%)	65% (of 89 cases with reported stage)
Tobacco use (%)	33% (of 76 cases with available smoking status)
Molecular testing performed (%)	58.3% (56/96)
Actionable genetic alterations (%)	58.9% (of tested cases)
ALK rearrangement (%)	32.9% (of tested cases)
EGFR mutation (%)	21.4% (of tested cases)
Received chemotherapy during pregnancy (%)	22.8% (22/96)
Gestational age at chemotherapy (mean, weeks)	21.8 ± 10.4

Percentages are calculated using the total cohort (N = 96) unless otherwise specified. For variables with incomplete reporting, percentages are calculated using the number of cases with available data, as indicated.

### Tumor characteristics and staging

Histologic subtype was reported in 93 cases (96.9%). The majority were non–small cell lung cancer, most commonly adenocarcinoma. Smaller numbers of squamous cell carcinoma, small cell lung cancer, and other rare histologies were also described.

Clinical stage at diagnosis was documented in 89 cases (92.7%). Approximately two-thirds of patients presented with stage III or IV disease, consistent with locally advanced or metastatic cancer at diagnosis. Early-stage disease was less common and was often identified incidentally or following imaging for persistent respiratory symptoms. Tumor characteristics and stage distribution are presented in **Table 2**.

### Molecular profiling

Molecular testing was reported in 56 cases (58.3% of the cohort). Among tested tumors, 33 (58.9%) harbored actionable driver alterations. ALK rearrangements were the most frequently reported (32.9%), followed by EGFR mutations (21.4%), with occasional reports of other targetable abnormalities. In exploratory analyses restricted to cases with available data, patients with identified driver alterations were more likely to receive targeted therapy in the postpartum period compared with those without reported alterations (p < 0.05). Notably, a subset of mutation-positive patients did not receive targeted therapy, most commonly due to rapid clinical deterioration, limited access, or the timing of diagnosis relative to the availability of these agents. Details of molecular testing and identified alterations are summarized in **Table 2**.

### Imaging and diagnostic workup

Nearly all patients underwent chest imaging as part of the diagnostic evaluation. Modalities included chest radiography and computed tomography, with magnetic resonance imaging and positron emission tomography used less frequently. In several cases, imaging was delayed or initially limited because of concerns regarding fetal radiation exposure, particularly in older reports. When ionizing radiation was used, imaging was generally performed with abdominal shielding and pregnancy-adapted protocols. No fetal complications were directly attributed to diagnostic imaging in the reported cases; however, this finding should be interpreted with caution, given the limited sample size and incomplete reporting in the available literature. Bronchoscopy and tissue biopsy were commonly used to establish the diagnosis and obtain material for histologic and molecular analysis.

### Systemic therapy during pregnancy

Systemic therapy during pregnancy was reported in 31 cases (32.3% of the cohort), with chemotherapy representing the predominant modality. Chemotherapy was administered during pregnancy in 22 cases (22.8%). Regimens most often consisted of platinum-based doublets, typically including cisplatin or carboplatin combined with etoposide, vinorelbine, or a taxane.

The mean gestational age at chemotherapy initiation was 21.8 weeks (standard deviation 10.4; range 12–34). In nearly all cases, treatment began during the second or third trimester, with first-trimester exposure reported rarely. Dosing was generally based on maternal body surface area, and treatment schedules were adjusted to avoid administration close to delivery. In most reports, chemotherapy was held for at least two to three weeks before planned delivery to reduce the risk of maternal and neonatal cytopenias. Radiotherapy during pregnancy was uncommon and was typically reserved for palliative indications, such as symptomatic brain or bone metastases. When used, careful planning was employed to minimize fetal exposure. Pregnancy and fetal outcomes stratified by chemotherapy exposure during pregnancy are summarized in **Table 3**.

**Table 3:** Pregnancy and fetal outcomes stratified by chemotherapy exposure during pregnancy.

Outcome	Overall cohort (N = 96)	Chemotherapy during pregnancy (n = 22)	No chemotherapy during pregnancy (n = 74)
Live birth	69 (71.9%)	15 (68.2%)	54 (73.0%)
Term delivery (≥37 weeks)	45 (46.9%)	9 (40.9%)	36 (48.6%)
Preterm delivery (<37 weeks)	24 (25.0%)	6 (27.3%)	18 (24.3%)
Pregnancy termination	17 (17.7%)	3 (13.6%)	14 (18.9%)
Fetal demise (intrauterine)	1 (1.0%)	0 (0%)	1 (1.4%)
Other reported outcomes*			
Spontaneous miscarriage	7 (7.3%)	1 (4.5%)	6 (8.1%)
Neonatal death	1 (1.0%)	0 (0%)	1 (1.4%)
Congenital anomalies reported	0 (0%)	0 (0%)	0 (0%)

Percentages are calculated within each subgroup using the number of cases with available data. Outcomes were variably reported across included case reports and case series, and the absence of reporting does not imply the absence of the outcome. Some outcome categories may overlap due to differences in reporting across studies. Comparisons between groups are descriptive only, as the heterogeneity of reporting and limited sample size preclude formal statistical inference.

### Postpartum treatment and targeted therapy

Following delivery, many patients received additional systemic therapy, including chemotherapy, targeted therapy, and, in more recent cases, immunotherapy.

Among the 33 patients with actionable driver alterations, 30 ultimately received targeted therapy, most commonly EGFR or ALK tyrosine kinase inhibitors. In nearly all cases, targeted agents were deferred until after delivery due to limited safety data during pregnancy.

Several patients experienced prolonged disease control after initiation of targeted therapy, particularly in more recent reports. However, follow-up duration varied widely, and survival outcomes were inconsistently reported. Postpartum treatment patterns are summarized in **Table 4**.

**Table 4:** Postpartum cancer-directed therapy among reported cases (N = 96).

Postpartum treatment modality	Patients, n (%)
Any postpartum systemic therapy	61 (63.5%)
Targeted therapy (TKIs)	30 (31.6%)
EGFR TKI	10 (10.4%)
ALK TKI	14 (14.6%)
Other targeted agents	6 (6.3%)
Immunotherapy (checkpoint inhibitors)	18 (18.8%)
Cytotoxic chemotherapy	25 (26.0%)
Surgery (postpartum lung resection)	12 (12.5%)
Radiotherapy	9 (9.4%)
Best supportive care only	11 (11.5%)

Postpartum therapy refers to cancer-directed treatment initiated after delivery. Percentages are calculated using the total cohort (N = 96). Percentages may exceed 100% because some patients received multiple treatment modalities. Targeted therapies were administered primarily in the postpartum setting, particularly among patients with reported actionable driver alterations (e.g., EGFR or ALK), reflecting current clinical practice in the absence of robust safety data during pregnancy.

### Pregnancy and fetal outcomes

Pregnancy outcomes were reported for all 96 cases. Among reported pregnancies, 69 (71.9%) resulted in live births. Term delivery occurred in 45 cases (46.9%), while preterm delivery occurred in 24 cases (25.0%). Pregnancy termination was reported in 17 cases (17.7%), and fetal demise in one case (1.0%). The remaining reports described ongoing pregnancies, incomplete outcome data, or outcomes that were not clearly specified.

Among live births with available information, most neonates had normal Apgar scores, and no major congenital anomalies were reported. Minor complications included low birth weight and transient neonatal cytopenias, particularly among infants exposed to chemotherapy during the third trimester.

Chemotherapy exposure during pregnancy was not associated with an increased rate of congenital malformations; however, this finding should be interpreted with caution, given the limited sample size, heterogeneity of reporting, and incomplete follow-up of neonatal outcomes.

In exploratory analyses restricted to cases with available outcome data, advanced lung cancer stage at diagnosis was significantly associated with adverse pregnancy outcomes, including preterm delivery, pregnancy termination, and fetal demise (p < 0.0001). Pregnancy and fetal outcomes stratified by chemotherapy exposure during pregnancy are summarized in **Table 3**.

### Placental and fetal pathology

Placental examination was reported in 39 cases (40.6%) and was performed selectively based on clinical context and reporting practices rather than routinely. Metastatic involvement of the placenta was identified in 5 cases (12.8% of examined placentas), corresponding to approximately 5% of the overall cohort.

All cases of placental metastasis occurred in the setting of advanced-stage maternal disease. No confirmed fetal metastatic involvement was reported. Infants born in cases with placental metastases were followed clinically, with no evidence of malignancy documented during the reported follow-up period. Placental and fetal pathology findings are summarized in **Table 5**.

**Table 5:** Placental and fetal pathology findings in reported cases.

Pathologic finding	Cases reported, n (%)
Placental histopathologic examination performed	39 (40.6%)
Placental metastasis identified	5 (12.8% of examined placentas)
Placental metastasis was not identified	34 (87.2% of examined placentas)
Fetal tissue examined	12 (12.5%)
Fetal metastatic involvement confirmed	0 (0%)
Umbilical cord involvement	0 (0%)
Amniotic fluid cytology performed	0 (0%)

Placental and fetal pathologic evaluation was not performed routinely and was reported selectively across included cases. Percentages are calculated using the total cohort (N = 96) unless otherwise specified. For placental metastasis, percentages are calculated among cases with reported placental examination. No cases of confirmed fetal metastatic involvement were identified among cases with available fetal or placental pathologic assessment.

### Maternal outcomes

Maternal outcomes were variably reported, with follow-up frequently limited, particularly in older case reports. Follow-up was defined as the interval from the time of diagnosis to the last reported follow-up or death, as documented in each case report. Survival outcomes were calculated descriptively using available follow-up data, with denominators varying according to reporting completeness across studies. Patients with advanced-stage disease often experience rapid progression and death within months of diagnosis. In contrast, patients diagnosed with earlier-stage disease who underwent surgical resection, with or without adjuvant therapy, generally demonstrated more favorable outcomes when follow-up data were available. Among patients with actionable driver alterations who received postpartum targeted therapy, several were reported to achieve survival beyond two years, with some cases describing ongoing disease control at last follow-up. However, the small number of cases, heterogeneity in reporting, and incomplete follow-up preclude definitive conclusions regarding long-term survival. Maternal outcomes are summarized in **Table 6**.

**Table 6:** Maternal survival and follow-up outcomes.

Outcome	Patients, n (%)
Maternal survival reported	78 (81.3% of cohort)
Median follow-up duration, months (IQR)	14 (6–28)
Alive at last reported follow-up	41 (52.6% of cases with reported survival)
Deceased at last reported follow-up	37 (47.4% of cases with reported survival)
Death attributed to lung cancer	35 (44.9% of cases with reported survival)
Death attributed to other causes	2 (2.6% of cases with reported survival)
Survival beyond 12 months	33 (42.3% of cases with reported follow-up)
Survival beyond 24 months	18 (23.1% of cases with reported follow-up)

Maternal survival and follow-up data were inconsistently reported across included studies. Percentages are calculated using the number of cases with available data for each outcome. Survival outcomes reflect the most recent follow-up reported in each case and should be interpreted with caution, given the heterogeneity in reporting and follow-up duration.

### Geographic distribution of reported cases

**Figure 3:**
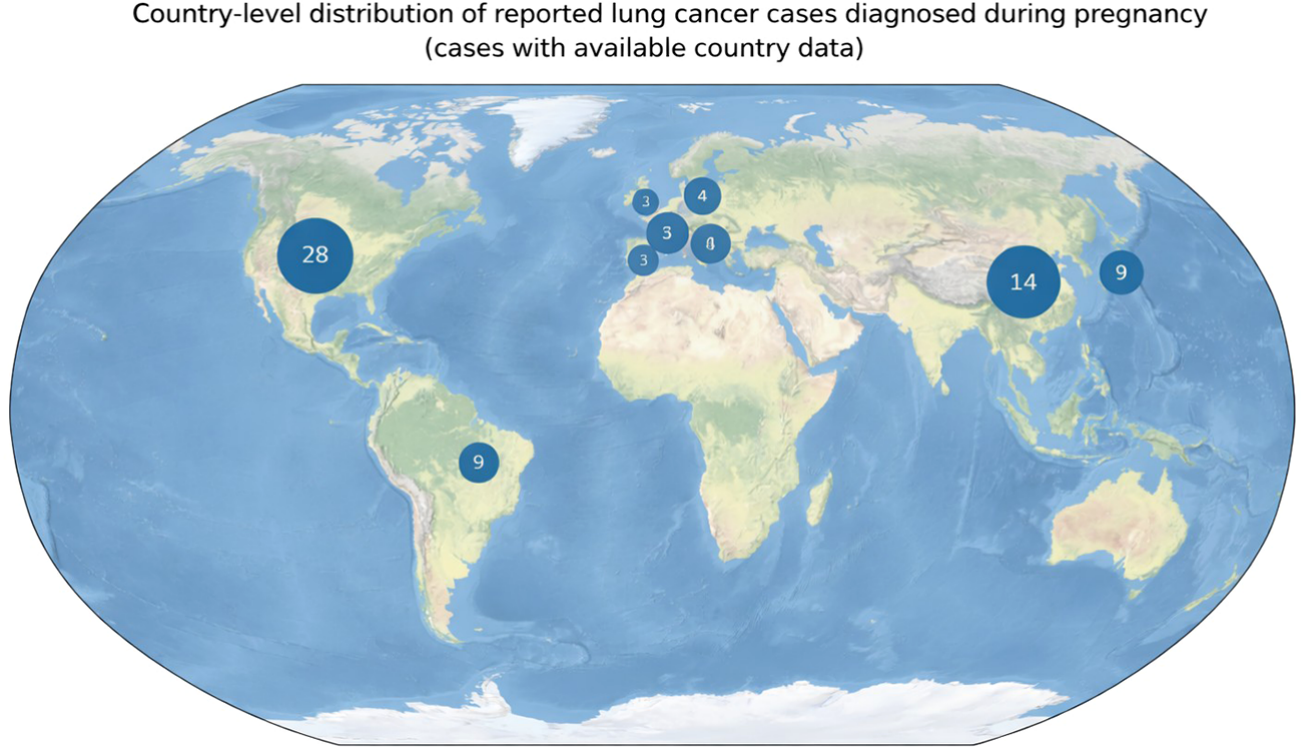
Country-level distribution of reported cases of lung cancer diagnosed during pregnancy. Circle size is proportional to the number of cases reported per country. Only cases with available country-level data (n = 79) are shown. The distribution reflects published cases and may be influenced by reporting practices and access to diagnostic and research resources.

Reported cases originated from multiple regions worldwide, with the highest numbers reported from North America, Europe, and East Asia. This distribution likely reflects both regional patterns of lung cancer incidence and differences in access to diagnostic resources and publication practices. To visually depict this pattern, a proportional symbol world map was generated (**Figure 3**), in which circle size corresponds to the number of reported cases per country. Only cases with available country-level data (n = 79) were included in this visualization. This approach highlights the concentration of published cases in a limited number of countries and underscores the probable underrepresentation of regions with fewer published reports, potentially reflecting reporting and publication bias.

## Discussion

This systematic review highlights both the rarity and the growing clinical relevance of lung cancer diagnosed during an ongoing pregnancy. Among the 96 cases identified, most patients presented with advanced-stage disease, a pattern that is consistent with prior reports suggesting that pregnant patients with lung cancer often experience diagnostic delays and modified diagnostic workup (5–10, 15, 31). These observations are also aligned with the broader literature on cancer during pregnancy, in which overlapping symptoms, concern for fetal safety, and limited familiarity with evolving guidance may contribute to later-stage diagnosis and more complex management decisions (1–3, 12, 14, 21).

Concerns regarding fetal radiation exposure continue to influence diagnostic decision-making in pregnant patients. Historically, these concerns have limited the use of chest CT, PET-CT, and other advanced imaging modalities in patients with suspected thoracic malignancy. Contemporary radiology and obstetric guidance, however, indicates that with appropriate shielding and pregnancy-adapted protocols, many imaging studies can be performed with fetal radiation doses well below thresholds associated with congenital malformations or pregnancy loss (12–15, 21). Despite this, pregnant patients with suspected lung cancer may still undergo delayed imaging and biopsy, which may contribute to the high proportion of stage III–IV disease observed in our cohort and in prior series (5–10, 15, 31). Together, these findings underscore the importance of disseminating clear, evidence-based imaging recommendations so that pregnancy itself does not become a barrier to timely cancer diagnosis.

Systemic therapy during pregnancy remains one of the most challenging aspects of management. Consistent with existing evidence and clinical guidelines, chemotherapy was rarely administered during the first trimester in our cohort, reflecting the established risk of major congenital anomalies associated with cytotoxic exposure during organogenesis (2, 16, 17). Instead, chemotherapy was most often delivered during the second and third trimesters, typically using platinum-based regimens. In our review, we did not identify a clear signal of major congenital malformations associated with chemotherapy administered later in pregnancy; however, this observation must be interpreted with caution, given the small sample size, heterogeneity of reporting, and lack of comprehensive long-term neonatal follow-up. Preterm delivery and low birth weight remained relatively common, and long-term neurodevelopmental outcomes were rarely reported (2, 18–23). Accordingly, our findings support cautious, individualized use of chemotherapy during the second and third trimesters when clinically necessary, within a multidisciplinary framework that carefully considers timing, regimen selection, and delivery planning.

Our review also highlights the importance of molecular profiling in contemporary lung cancer care and its relevance in pregnancy-associated disease. Among patients who underwent molecular testing, more than half had actionable driver alterations, most commonly EGFR mutations and ALK rearrangements, consistent with the known enrichment of these alterations in younger, often never-smoking patients (10, 24–31). At the same time, this proportion should be interpreted in the context of temporal changes in practice, as many earlier published cases predated the routine use of molecular testing and likely underestimate the true prevalence of actionable alterations in this population. This molecular landscape is clinically important because targeted therapies have become the standard of care in the nonpregnant population, offering superior response rates and progression-free survival in appropriately selected patients (25–30).

In our cohort, targeted therapies were predominantly initiated in the postpartum period, with only a small number of patients receiving tyrosine kinase inhibitors (TKIs) during pregnancy after careful multidisciplinary deliberation. This pattern likely reflects the absence of robust safety data, concern regarding placental transfer, and uncertainty regarding fetal toxicity (21, 31, 32). Available evidence on the use of TKIs in pregnancy remains limited to isolated case reports and small observational experiences (32). As a result, routine practice will likely continue to favor deferring targeted therapy until after delivery, when feasible, while ensuring that molecular profiling is obtained promptly so that postpartum treatment can be initiated without unnecessary delay.

Immunotherapy warrants separate consideration given its central role in modern thoracic oncology and the limited data available in pregnancy. Immune checkpoint inhibitors have transformed the management of advanced non–small cell lung cancer in the nonpregnant setting (26), but their use during pregnancy remains poorly characterized. From a biological standpoint, the PD-1/PD-L1 axis contributes to maternal–fetal immune tolerance, raising theoretical concerns that immune checkpoint blockade could disrupt placental immune homeostasis and adversely affect fetal development (21). Human data remain sparse and are largely limited to isolated case reports, extrapolation from other malignancies, and pharmacovigilance observations (21, 32). Accordingly, immunotherapy should be approached with extreme caution in pregnancy, and in most circumstances, deferral until the postpartum period remains the most prudent strategy.

Our analysis of placental and fetal outcomes provides cautious reassurance but should not be overinterpreted. Placental pathology was reported in a minority of cases and was performed selectively rather than routinely, with metastatic involvement identified in a small subset. No confirmed cases of fetal metastasis were identified. These findings are broadly consistent with prior reports suggesting that although placental involvement may occur, particularly in the setting of advanced maternal disease, direct fetal involvement remains rare (33–35). However, the available data are limited and heterogeneous, and the absence of reported fetal metastasis should not be interpreted as proof of absence of risk. These observations support consideration of placental examination and neonatal follow-up, particularly in cases of advanced-stage maternal disease.

Several broader considerations arise from this review, but should be interpreted as contextual rather than direct findings of our dataset. Prior population-based studies have shown that Black and Hispanic patients are more likely than White patients to present with advanced-stage lung cancer and less likely to receive molecular testing or guideline-concordant therapy (33–36). Structural factors, including insurance status, geographic access to specialized centers, language barriers, and systemic bias, likely contribute to these differences (33–40). Although our review was not designed to evaluate disparities directly, such factors may plausibly influence care delivery in pregnancy-associated lung cancer as well. Similarly, patient-centered care, including attention to motherhood, survivorship, communication, and values-based decision-making, remains highly relevant in this setting (37, 38). We have therefore framed these issues as important contextual considerations rather than conclusions directly derived from the pooled cases in this review.

Our findings support a model of care in which pregnant patients with lung cancer are managed by coordinated multidisciplinary teams that include thoracic oncology, maternal–fetal medicine, obstetrics, neonatology, radiology, pathology, palliative care, and social work. Such teams can help navigate the timing of imaging, biopsy, systemic therapy, and delivery; ensure that molecular profiling is not overlooked; and incorporate the patient’s values and goals into shared decision-making. Recent expert commentaries have advocated for more formal “onco-obstetrics” pathways that bridge oncology and obstetrics (11, 21). Our review supports the importance of such integrated frameworks, particularly as targeted therapies and immunotherapies become increasingly embedded in routine thoracic oncology practice.

This review has several important limitations. The evidence base consists almost entirely of case reports and small case series and is therefore highly vulnerable to publication bias, reporting bias, and incomplete follow-up. Cases with unusual molecular features, favorable outcomes, or striking treatment responses may be more likely to be published, whereas negative or less eventful experiences may remain underreported. Many reports lacked standardized staging, detailed treatment information, and systematic long-term follow-up of both mothers and children. In addition, the literature spans multiple therapeutic eras, complicating comparisons across time and likely contributing to heterogeneity in diagnostic evaluation, molecular testing, and treatment availability. Our exploratory statistical analyses were based on limited case-level data, with variable denominators and incomplete reporting, and should therefore be interpreted as hypothesis-generating rather than confirmatory. Likewise, the survival summaries presented in this review reflect the most recent follow-up available in each report and should not be interpreted as directly comparable survival estimates across cases.

Despite these limitations, this study provides one of the most comprehensive syntheses to date of lung cancer diagnosed during pregnancy, integrating clinical, molecular, treatment, and maternal–fetal outcome data across several decades. The high proportion of advanced-stage presentation, the frequent identification of actionable driver alterations among tested tumors, the predominant postpartum use of targeted therapy, and the association between advanced maternal stage and adverse pregnancy outcomes provide a useful descriptive framework for contemporary clinical practice. At the same time, the limited evidence base and inconsistent outcome reporting underscore the need for prospective, multicenter registries that systematically capture maternal, obstetric, placental, neonatal, and long-term child outcomes alongside detailed molecular and treatment data. Such efforts will be essential to inform future guidance and improve care for this rare but clinically consequential condition.

## Conclusion

Lung cancer during pregnancy remains a rare but clinically complex scenario that requires timely diagnosis and coordinated multidisciplinary care. In this systematic review, most patients presented with advanced-stage disease, underscoring the potential impact of diagnostic delays and the importance of appropriate use of imaging and biopsy during pregnancy. Chemotherapy administered during the second and third trimesters was feasible in selected cases. Within the limitations of this case-based literature, no clear signal of major congenital toxicity was identified; however, these findings should be interpreted with caution given the small sample size, heterogeneity of reporting, and lack of long-term follow-up. A high prevalence of actionable molecular alterations was observed among tested tumors, most commonly ALK rearrangements and EGFR mutations. Targeted therapies were predominantly initiated in the postpartum period, reflecting current clinical practice in the absence of robust safety data during pregnancy. This further underscores the importance of timely molecular profiling to guide treatment planning. Overall, these findings support a model of care centered on early evaluation, routine molecular testing, and multidisciplinary decision-making. The development of prospective registries and collaborative, multicenter research efforts will be essential to generate higher-quality evidence and improve outcomes for both mothers and their children in this rare but high-stakes clinical setting.

## Data Availability

Publicly available datasets were analyzed in this study. This study is a systematic review of published case reports and case series. No new datasets were generated or deposited. Case-level data were extracted from published sources for the purpose of qualitative and descriptive synthesis. Due to the nature of the source material, individual case-level datasets are not publicly shared. All primary sources are cited in the manuscript, and further details are available from the corresponding author upon reasonable request.
