# Supplementary material for: Treating without guidelines: management and outcomes of lung cancer diagnosed during pregnancy — a systematic review
Source: Front Oncol. 2026 May 13;16:1780320. doi: 10.3389/fonc.2026.1780320 (PMC13212082; doi:10.3389/fonc.2026.1780320)
Supplement: Supplementary file 1 [file Table1.docx]

**Supplementary Table S1. Electronic Search Strategies**

| **Database** | **Search Strategy** | **Limits / Notes** |
| --- | --- | --- |
| **PubMed / MEDLINE** | (("Lung Neoplasms"[Mesh] OR "lung cancer" OR "pulmonary cancer" OR "non-small cell lung cancer" OR NSCLC OR "small cell lung cancer" OR SCLC) AND ("Pregnancy"[Mesh] OR pregnancy OR pregnant OR gestation OR maternal)) | Humans; English language |
| **Embase** | ('lung cancer'/exp OR 'lung neoplasm*' OR 'pulmonary cancer' OR 'non small cell lung cancer' OR nsclc OR 'small cell lung cancer' OR sclc) AND ('pregnancy'/exp OR pregnancy OR pregnant OR gestation OR maternal) | Human studies: articles, case reports, case series |
| **Scopus** | TITLE-ABS-KEY(("lung cancer" OR "lung neoplasm*" OR "pulmonary cancer" OR "non-small cell lung cancer" OR NSCLC OR "small cell lung cancer" OR SCLC) AND (pregnancy OR pregnant OR gestation OR maternal)) | No language restriction at the search stage |
| **Google Scholar** | ("lung cancer" OR "pulmonary malignancy") AND (pregnancy OR pregnant) | First 300 results screened manually; reference lists hand-searched |

*Search strategies were adapted for each database using controlled vocabulary and free-text terms. Boolean operators and truncation were applied as appropriate. Searches were designed to maximize sensitivity given the rarity of lung cancer diagnosed during pregnancy.*
